# Septins As Modulators of Endo-Lysosomal Membrane Traffic

**DOI:** 10.3389/fcell.2016.00124

**Published:** 2016-11-03

**Authors:** Kyungyeun Song, Giulia Russo, Michael Krauss

**Affiliations:** Leibniz-Institut für Molekulare PharmakologieBerlin, Germany

**Keywords:** septins, membrane, phosphoinositides, endocytosis, endosome, sorting

## Abstract

Septins constitute a family of GTP-binding proteins, which assemble into non-polar filaments in a nucleotide-dependent manner. These filaments can be recruited to negatively charged membrane surfaces. When associated with membranes septin filaments can act as diffusion barriers, which confine subdomains of distinct biological functions. In addition, they serve scaffolding roles by recruiting cytosolic proteins and other cytoskeletal elements. Septins have been implicated in a large variety of membrane-dependent processes, including cytokinesis, signaling, cell migration, and membrane traffic, and several family members have been implicated in disease. However, surprisingly little is known about the molecular mechanisms underlying their biological functions. This review summarizes evidence in support of regulatory roles of septins during endo-lysosomal sorting, with a particular focus on phosphoinositides, which serve as spatial landmarks guiding septin recruitment to distinct subcellular localizations.

## Introduction

Septins constitute a family of small GTPases that assemble into filaments and higher-order structures in a nucleotide-dependent manner (Kinoshita, 2003). In humans 13 different paralogs are expressed in a cell- and tissue-specific manner, which have been classified into the SEPT2, SEPT3, SEPT6, and SEPT7 subgroups based on sequence similarity. All septins share a central GTPase domain that allows for their oligomerization into filaments (Sirajuddin et al., 2007). The G-domain is flanked by more variable N- and C-terminal extensions, which provide interfaces for the association with non-septin binding partners. Septins have been implicated in a large variety of membrane-dependent processes, including cytokinesis, signaling and membrane traffic. During these events they act as barriers limiting diffusion of membrane-resident factors, but also as molecular scaffolds that orchestrate the recruitment of downstream effectors (Caudron and Barral, 2009). Given the fundamental importance of septin-dependent processes, it is not surprising that several septin family members have been implicated in disease, such as Alzheimer's disease or cancer (Dolat et al., 2014). However, surprisingly little is known about the molecular mechanisms underlying septin-related pathogenesis.

## Septin filaments associate with membrane surfaces

Septins bind to membrane surfaces enriched in negatively charged phospholipids, in particular phosphoinositides (PIs) (Zhang et al., 1999; Tanaka-Takiguchi et al., 2009). This is mediated by a patch of basic amino acids found in members of the SEPT2, SEPT3, and SEPT7 subgroups, which is located in close proximity to the G-domain. Because septins assemble into hetero-oligomeric filaments the association with negatively charged membrane surfaces might be a cooperative mechanism facilitated by septin oligomerization. *Vice versa*, it has been noted that membrane association supports the assembly of septins into filaments (Bridges et al., 2014). In the best-characterized septin complex SEPT2/SEPT6/SEPT7 a flexible region at the SEPT2 dimer interface promotes bending of the oligomer (Sirajuddin et al., 2007). This might explain why SEPT2-6-7 filaments can impose positive membrane curvature (Tanaka-Takiguchi et al., 2009). More recently Bridges et al. demonstrated that septins also associate with lipid bilayers devoid of negatively charged lipid species, and in this case sense micron-scale, positive membrane curvature *in vitro* and in living cells (Bridges et al., 2016).

Both, the generation and the recognition of membrane curvature are hallmarks of proteins involved in intracellular membrane traffic (Krauss and Haucke, 2011). Membrane traffic relies on the formation of transport carriers, which depends on the concentration of cargo in confined membrane areas. Furthermore, select machineries need to be assembled to aid membrane deformation into highly curved vesicles, and to promote fission from the donor compartment. Given the proposed scaffolding function of septin filaments, and their well-established roles as diffusion barriers, it is conceivable that septins contribute to carrier formation, for instance by shielding membrane subdomains, by imposing or sensing membrane curvature, or by recruiting appropriate effector proteins assisting vesicle generation.

Septins have been shown to bind to a variety of PI species, which are generated on distinct subcellular membranes (see below for details), and might thus act during carrier formation at different organelles. This is supported by several studies that identified septin binding partners with functions during endo-lysosomal sorting (Table 1).

**Table 1:** **Binding partners of mammalian septin family members with implication in endo-lysosomal sorting**.

**Septin**	**Binding partner**	**Process**	**References**
SEPT3	GABA-RAPL2	Autophagy	Nakahira et al., 2010
SEPT3	SNX6	Endosomal sorting	
SEPT3	Myo1b	Endosomal sorting	
SEPT8	RALBP1	Endocytosis, endosomal sorting	
SEPT9	CIN85 (SH3KBP1)	Endosomal sorting	
SEPT8	BLOC-1	Endosomal sorting	Gokhale et al., 2012
SEPT5/SEPT11	Dynamin	Endocytosis	Maimaitiyiming et al., 2013
SEPT7	AP-3	Endosomal sorting	Traikov et al., 2014
SEPT9	CIN85 (SH3KBP1)	Endosomal sorting	Diesenberg et al., 2015

## Septins regulate the formation of endocytic carriers at the plasma membrane

Membrane recruitment of septin filaments is supported by PI(4,5)P_2_ and PI(3,4,5)P_3_, two PI species found predominantly at the plasma membrane (Zhang et al., 1999). Depletion of plasmalemmal PI(4,5)P_2_ disrupts the integrity of septin filaments, indicating that this PI orchestrates filament assembly. Moreover, septins have been suggested to support the generation of PI(4,5)P_2_-enriched microdomains at the plasma membrane, and to thereby promote the formation of junctions transiently formed with the endoplasmic reticulum during store-operated calcium entry (Sharma et al., 2013). This suggests that septin filaments can undergo dynamic re-organization in a spatiotemporally regulated manner, together with PI(4,5)P_2_ pools at the plasma membrane.

**Figure 1:**
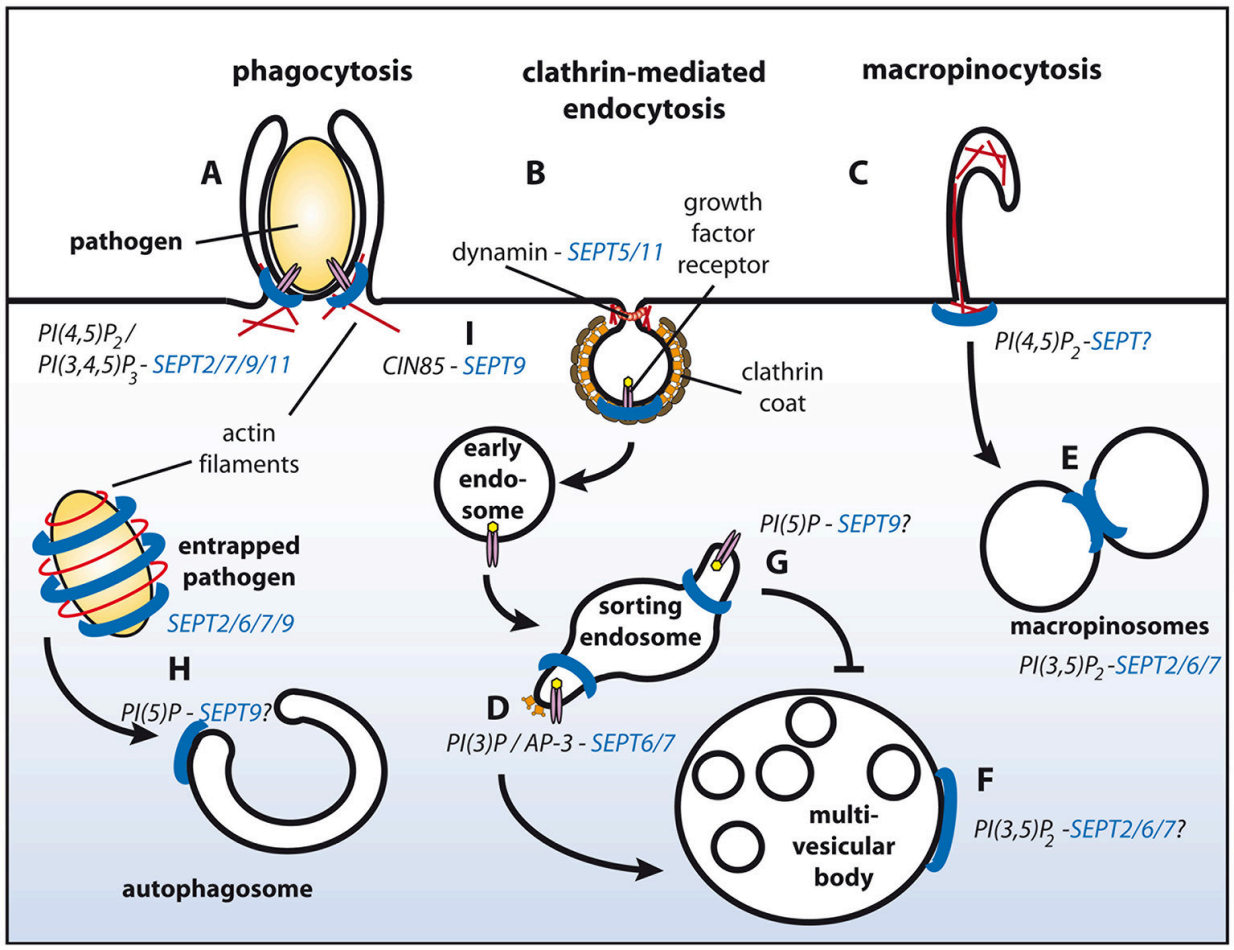
**Schematic representation of endosomal sorting events with proven or putative involvement of septins**. Septin filaments are indicated in blue, actin filaments in red. SEPT2/7/9/11 promote phagocytosis **(A)**. Through associating with dynamin SEPT5/11 might function during clathrin-mediated endocytosis **(B)**. Interactions of septins with actin filaments might modulate macropinocytosis **(C)**. Complex formation between AP-3 and SEPT6/7 facilitates degradative sorting **(D)**. SEPT2/6/7 promote fusion of macropinosomes **(E)** and potentially of MVBs with lysosomes **(F)**. PI(5)P at endosomes protects cargo from degradative sorting, and might recruit SEPT9 to sorting endosomes **(G)**. SEPT2/6/7/9 form cages around intracellular pathogens and promote their sorting to autophagosomes **(H)**. SEPT9 inhibits ubiquitylation of EGF receptors at the plasma membrane by associating with CIN85, and thereby attenuates degradative sorting at endosomes **(I)**. See text for details.

The generation of PI(4,5)P_2_ initiates many plasma membrane-derived processes, including endocytosis (Krauss and Haucke, 2007), and indeed, several studies implicated septins in endocytic events. Depletion of SEPT2 or SEPT11 in macrophages perturbs phagocytic uptake of opsonized latex beads (Huang et al., 2008), and at sites of phagosome formation both septin family members co-localize with actin-rich structures, at a time when PI(4,5)P_2_ accumulates at the same spots (Figure 1A). Similarly, several septins have been found to assemble in close proximity to actin at the entry site of several pathogens (i.e., *Listeria* and *Candida*) in human non-phagocytic cells, and have been proven important for their internalization (Mostowy and Cossart, 2011; Phan et al., 2013).

Interestingly, entry of some pathogens additionally depends on parts of the clathrin machinery, including clathrin itself, but also the vesicle fission enzyme dynamin, and a number of accessory proteins known to associate with receptor tyrosine kinases during their endocytosis (Veiga and Cossart, 2006). Some of these factors encode SH3-domains, which could potentially interact with proline-rich stretches present in several septins. One of them, CIN85/SH3KBP1, interacts with SEPT9 (Diesenberg et al., 2015) (see below for details) and could thereby link the clathrin machinery to SEPT9-containing filaments. Based on these findings it is tempting to speculate that septins also participate in other actin- and/ or dynamin-dependent endocytic pathways, such as clathrin-mediated and caveolar endocytosis, or macropinocytosis (Figures 1B,C). In line with this hypothesis SEPT5 and SEPT11 are found in complexes with dynamin (Maimaitiyiming et al., 2013). Furthermore, yeast septins associate with a subset of endocytic proteins (Renz et al., 2016), including the dynamin-like GTPase Vps1, and the accessory proteins Sla2 (ortholog of the mammalian clathrin- and actin binding protein Hip1R) and Syp1 (ortholog of mammalian FCHo proteins that are believed to nucleate clathrin coat formation at the plasma membrane).

## Septins during endosomal sorting

Accumulating evidence suggests that septins also associate with membranes of the endo-lysosomal system. A proteomic approach identified several septins together with *bona fide* endosomal proteins on early endosome-like liposomes containing PI(3)P (Baust et al., 2008). Later, it was demonstrated that SEPT6 and SEPT7 on endosomes regulate the biogenesis of multivesicular bodies (MVBs) in a process involving the adaptor complex AP-3 and ESCRT proteins (Traikov et al., 2014). They thereby facilitate the degradation of ubiquitylated cargo proteins in lysosomes (Figure 1D).

During maturation into MVBs PI(3)P-positive endosomes acquire a PI(3,5)P_2_-positive identity, which is generated by FYVE finger containing PI kinase (PIKfyve) to promote endo-lysosomal fusion. Interestingly, a PIKfyve-dependent pool of PI(3,5)P_2_ recruits SEPT2 to fusion sites on Rab7-positive macropinosomes (Dolat and Spiliotis, 2016; Figure 1E). SEPT2 depletion does not impair docking between macropinosomes, but reduces their fusion. It remains elusive if this defect is caused by a direct modulation of the SNARE machinery through SEPT2. Alternatively, SEPT2-containing filaments might directly confer fusogenic properties. Whether SEPT2 is recruited to MVBs as well, is currently unknown (Figure 1F).

Several physiological stimuli, but also infection with certain pathogens, can up-regulate PI(5)P on endosomes. In cells infected with *Shigella* this pool of endosomal PI(5)P impedes EGF receptor degradation (Ramel et al., 2011; Boal et al., 2015). Interestingly, exogenous supply of PI(5)P translocates SEPT9 to lipid droplets and possibly other organelles (Akil et al., 2016; Figure 1G).

Besides its role at endosomes, PI(5)P can also regulate the biogenesis of autophagosomes through a non-canonical, Vps34-independent pathway (Vicinanza et al., 2015). Interestingly, it has been noted that SEPT9 and SEPT7 are incorporated into septin cages that entrap cytosolic *Shigella* to target them for autophagy (Mostowy et al., 2010; Sirianni et al., 2016; Figure 1H). Septin cage formation occurs in concert with, and dependent on proteins involved in autophagy, including p62/SQSTM1, Atg5, Atg6, and Atg7. It is, thus, tempting to speculate that septins have a regulatory role during autophagy.

## Indirect effects of septins on degradative sorting of receptor tyrosine kinases

Septins can also exert indirect effects on sorting of cargo proteins. We recently noted a profound decrease in surface levels of epidermal growth factor (EGF) receptors upon depletion of SEPT9 (Diesenberg et al., 2015; Figure 1I). This effect depends on a proline-rich motif within the SEPT9 N-terminal domain that supports its association with the adaptor protein CIN85/SH3KBP1. CIN85-SEPT9 complexes localize exclusively to the plasma membrane, where SEPT9 is recruited to ligand-engaged receptors in a CIN85-dependent manner. CIN85 promotes down-regulation of EGF receptors through its interaction with the ubiquitin ligase Cbl (Soubeyran et al., 2002). As SEPT9 competes with Cbl for the same binding sites on CIN85 it negatively regulates receptor multi-ubiquitylation and thereby attenuates subsequent degradative sorting of ubiquitylated EGF receptors to lysosomes.

Similar mechanisms might apply for other receptors down-regulated by the CIN85/Cbl module, such as the hepatoctyte growth factor Met (Petrelli et al., 2002). In support of this hypothesis, decreased levels of Met have been detected in cells depleted of septins (Mostowy et al., 2011). As Met serves as a docking site for *Listeria*, this might provide an additional explanation for the reduced capability of this pathogen to invade host cells in absence of septins.

Marcus et al. have reported recently that septin oligomerization stabilizes ErbB2 (Marcus et al., 2016), a receptor tyrosine kinase mutated or overexpressed in multiple cancers. SEPT2 and SEPT9 co-localize with Erb2 at the basolateral plasma membrane of gastric cancer cells. Treatment of cells with forchlorfenuron, an inhibitor impairing septin assembly and dynamics, as well as septin depletion aggravate ubiquitin-dependent degradation of ErbB2, similar to what has been seen for EGF receptors. However, as ErbB2 is sorted independently of the CIN85/Cbl-module, alternative effectors apparently act downstream of septins in this case.

## Concluding remarks

Septins have been found to associate with a variety of PIs at different intracellular membranes, where they regulate a variety of cellular processes. As outlined above individual septin family members can thereby exert distinct effects, depending on their subcellular localizations. This is exemplified by SEPT2, which is recruited to the plasma membrane, to endosomal membranes or to the surface of mitochondria, where it controls unique events (the formation of phagocytic carriers, endosomal membrane fusion or organelle fission, respectively) (Huang et al., 2008; Mostowy and Cossart, 2011; Phan et al., 2013; Dolat and Spiliotis, 2016; Pagliuso et al., 2016). Thus, specificity in SEPT2 membrane recruitment and function must be accomplished through additional factors. This could be other septin family members that assemble with SEPT2 into filaments of distinct compositions, thereby conferring unique PI specificities. The association of filaments with organelle-specific, non-septin binding partners might generate additional flexibility in membrane targeting. Future studies will need to carefully dissect the exact composition of septin scaffolds to allow for a detailed understanding of their functions in endo-lysosomal sorting. Finally, the fact that the application of a septin inhibitor can counteract the stabilization of signaling receptors at the plasma membrane in cancer cells may offer an avenue for the treatment of cancer, and potentially other septin-related diseases.

## Author contributions

KS, GR, and MK participated in outlining and writing this Minireview. MK prepared figure and table.

## Funding

This work was supported by a grant of from the German research funding agency Deutsche Forschungsgemeinschaft (grant number SFB958/A11).

### Conflict of interest statement

The authors declare that the research was conducted in the absence of any commercial or financial relationships that could be construed as a potential conflict of interest.

## References

[B1] Akil A. Peng J. Omrane M. Gondeau C. Desterke C. Marin M.. (2016). Septin 9 induces lipid droplets growth by a phosphatidylinositol-5-phosphate and microtubule-dependent mechanism hijacked by HCV. Nat. Commun. 7:12203. 10.1038/ncomms12203 27417143 PMC4947189

[B2] Baust T. Anitei M. Czupalla C. Parshyna I. Bourel L. Thiele C.. (2008). Protein networks supporting AP-3 function in targeting lysosomal membrane proteins. Mol. Biol. Cell 19, 1942–1951. 10.1091/mbc.E08-02-0110 18287518 PMC2366865

[B3] Boal F. Mansour R. Gayral M. Saland E. Chicanne G. Xuereb J. M.. (2015). TOM1 is a PI5P effector involved in the regulation of endosomal maturation. J. Cell Sci. 128, 815–827. 10.1242/jcs.166314 25588840

[B4] Bridges A. A. Jentzsch M. S. Oakes P. W. Occhipinti P. Gladfelter A. S. (2016). Micron-scale plasma membrane curvature is recognized by the septin cytoskeleton. J. Cell Biol. 213, 23–32. 10.1083/jcb.201512029 27044896 PMC4828694

[B5] Bridges A. A. Zhang H. Mehta S. B. Occhipinti P. Tani T. Gladfelter A. S. (2014). Septin assemblies form by diffusion-driven annealing on membranes. Proc. Natl. Acad. Sci. U.S.A. 111, 2146–2151. 10.1073/pnas.1314138111 24469790 PMC3926015

[B6] Caudron F. Barral Y. (2009). Septins and the lateral compartmentalization of eukaryotic membranes. Dev. Cell 16, 493–506. 10.1016/j.devcel.2009.04.003 19386259

[B7] Diesenberg K. Beerbaum M. Fink U. Schmieder P. Krauss M. (2015). SEPT9 negatively regulates ubiquitin-dependent downregulation of EGFR. J. Cell Sci. 128, 397–407. 10.1242/jcs.162206 25472714

[B8] Dolat L. Hu Q. Spiliotis E. T. (2014). Septin functions in organ system physiology and pathology. Biol. Chem. 395, 123–141. 10.1515/hsz-2013-0233 24114910 PMC4452026

[B9] Dolat L. Spiliotis E. T. (2016). Septins promote macropinosome maturation and traffic to the lysosome by facilitating membrane fusion. J. Cell Biol. 214, 517–527. 10.1083/jcb.201603030 27551056 PMC5004444

[B10] Gokhale A. Larimore J. Werner E. So L. Moreno-De-Luca A. Lese-Martin C.. (2012). Quantitative proteomic and genetic analyses of the schizophrenia susceptibility factor dysbindin identify novel roles of the biogenesis of lysosome-related organelles complex 1. J. Neurosci. 32, 3697–3711. 10.1523/JNEUROSCI.5640-11.2012 22423091 PMC3313842

[B11] Huang Y. W. Yan M. Collins R. F. Diciccio J. E. Grinstein S. Trimble W. S. (2008). Mammalian septins are required for phagosome formation. Mol. Biol. Cell 19, 1717–1726. 10.1091/mbc.E07-07-0641 18272790 PMC2291437

[B12] Kinoshita M. (2003). Assembly of mammalian septins. J. Biochem. 134, 491–496. 10.1093/jb/mvg182 14607974

[B13] Krauss M. Haucke V. (2007). Phosphoinositides: regulators of membrane traffic and protein function. FEBS Lett. 581, 2105–2111. 10.1016/j.febslet.2007.01.089 17316616

[B14] Krauss M. Haucke V. (2011). Shaping membranes for endocytosis. Rev. Physiol. Biochem. Pharmacol. 161, 45–66. 10.1007/112_2008_2 22128406

[B15] Maimaitiyiming M. Kobayashi Y. Kumanogoh H. Nakamura S. Morita M. Maekawa S. (2013). Identification of dynamin as a septin-binding protein. Neurosci. Lett. 534, 322–326. 10.1016/j.neulet.2012.12.002 23260429

[B16] Marcus E. A. Tokhtaeva E. Turdikulova S. Capri J. Whitelegge J. P. Scott D. R.. (2016). Septin oligomerization regulates persistent expression of ErbB2/HER2 in gastric cancer cells. Biochem. J. 473, 1703–1718. 10.1042/BCJ20160203 27048593 PMC4903893

[B17] Mostowy S. Bonazzi M. Hamon M. A. Tham T. N. Mallet A. Lelek M.. (2010). Entrapment of intracytosolic bacteria by septin cage-like structures. Cell Host Microbe 8, 433–444. 10.1016/j.chom.2010.10.009 21075354

[B18] Mostowy S. Cossart P. (2011). Septins as key regulators of actin based processes in bacterial infection. Biol. Chem. 392, 831–835. 10.1515/BC.2011.078 21749282

[B19] Mostowy S. Janel S. Forestier C. Roduit C. Kasas S. Pizarro-Cerdá J.. (2011). A role for septins in the interaction between the *Listeria* monocytogenes INVASION PROTEIN InlB and the Met receptor. Biophys. J. 100, 1949–1959. 10.1016/j.bpj.2011.02.040 21504731 PMC3077699

[B20] Nakahira M. Macedo J. N. Seraphim T. V. Cavalcante N. Souza T. A. Damalio J. C.. (2010). A draft of the human septin interactome. PLoS ONE 5:e13799. 10.1371/journal.pone.0013799 21082023 PMC2970546

[B21] Pagliuso A. Tham T. N. Stevens J. K. Lagache T. Persson R. Salles A.. (2016). A role for septin 2 in Drp1-mediated mitochondrial fission. EMBO Rep. 17, 858–873. 10.15252/embr.201541612 27215606 PMC5278612

[B22] Petrelli A. Gilestro G. F. Lanzardo S. Comoglio P. M. Migone N. Giordano S. (2002). The endophilin-CIN85-Cbl complex mediates ligand-dependent downregulation of c-Met. Nature 416, 187–190. 10.1038/416187a 11894096

[B23] Phan Q. T. Eng D. K. Mostowy S. Park H. Cossart P. Filler S. G. (2013). Role of endothelial cell septin 7 in the endocytosis of *Candida albicans*. MBio 4, e00542–13. 10.1128/mBio.00542-13 24345743 PMC3870263

[B24] Ramel D. Lagarrigue F. Pons V. Mounier J. Dupuis-Coronas S. Chicanne G.. (2011). Shigella flexneri infection generates the lipid PI5P to alter endocytosis and prevent termination of EGFR signaling. Sci Signal 4, ra61. 10.1126/scisignal.2001619 21934107

[B25] Renz C. Oeljeklaus S. Grinhagens S. Warscheid B. Johnsson N. Gronemeyer T. (2016). Identification of cell cycle dependent interaction partners of the septins by quantitative mass spectrometry. PLoS ONE 11:e0148340. 10.1371/journal.pone.0148340 26871441 PMC4752459

[B26] Sharma S. Quintana A. Findlay G. M. Mettlen M. Baust B. Jain M.. (2013). An siRNA screen for NFAT activation identifies septins as coordinators of store-operated Ca^2+^ entry. Nature 499, 238–242. 10.1038/nature12229 23792561 PMC3846693

[B27] Sirajuddin M. Farkasovsky M. Hauer F. Kühlmann D. Macara I. G. Weyand M.. (2007). Structural insight into filament formation by mammalian septins. Nature 449, 311–315. 10.1038/nature06052 17637674

[B28] Sirianni A. Krokowski S. Lobato-Márquez D. Buranyi S. Pfanzelter J. Galea D.. (2016). Mitochondria mediate septin cage assembly to promote autophagy of Shigella. EMBO Rep. 17, 1029–1043. 10.15252/embr.201541832 27259462 PMC4931556

[B29] Soubeyran P. Kowanetz K. Szymkiewicz I. Langdon W. Y. Dikic I. (2002). Cbl-CIN85-endophilin complex mediates ligand-induced downregulation of EGF receptors. Nature 416, 183–187. 10.1038/416183a 11894095

[B30] Tanaka-Takiguchi Y. Kinoshita M. Takiguchi K. (2009). Septin-mediated uniform bracing of phospholipid membranes. Curr. Biol. 19, 140–145. 10.1016/j.cub.2008.12.030 19167227

[B31] Traikov S. Stange C. Wassmer T. Paul-Gilloteaux P. Salamero J. Raposo G.. (2014). Septin6 and Septin7 GTP binding proteins regulate AP-3- and ESCRT-dependent multivesicular body biogenesis. PLoS ONE 9:e109372. 10.1371/journal.pone.0109372 25380047 PMC4224394

[B32] Veiga E. Cossart P. (2006). The role of clathrin-dependent endocytosis in bacterial internalization. Trends Cell Biol. 16, 499–504. 10.1016/j.tcb.2006.08.005 16962776 PMC7126422

[B33] Vicinanza M. Korolchuk V. I. Ashkenazi A. Puri C. Menzies F. M. Clarke J. H.. (2015). PI(5)P regulates autophagosome biogenesis. Mol. Cell 57, 219–234. 10.1016/j.molcel.2014.12.007 25578879 PMC4306530

[B34] Zhang J. Kong C. Xie H. McPherson P. S. Grinstein S. Trimble W. S. (1999). Phosphatidylinositol polyphosphate binding to the mammalian septin H5 is modulated by GTP. Curr. Biol. 9, 1458–1467. 10.1016/S0960-9822(00)80115-3 10607590

